# Major Tumor Suppressor and Oncogenic Non-Coding RNAs: Clinical Relevance in Lung Cancer

**DOI:** 10.3390/cells6020012

**Published:** 2017-05-09

**Authors:** Kentaro Inamura

**Affiliations:** Division of Pathology, The Cancer Institute, Japanese Foundation for Cancer Research, 3-8-31 Ariake, Koto-ku, Tokyo 135-8550, Japan; kentaro.inamura@jfcr.or.jp; Tel.: +81-3-3570-0111 (ext. 5604); Fax: +81-3-3570-0558

**Keywords:** adenocarcinoma, carcinoma, carcinogenesis, histology, long non-coding RNA, miRNA, molecular pathology, oncology

## Abstract

Lung cancer is the leading cause of cancer deaths worldwide, yet there remains a lack of specific and sensitive tools for early diagnosis and targeted therapies. High-throughput sequencing techniques revealed that non-coding RNAs (ncRNAs), e.g., microRNAs and long ncRNAs (lncRNAs), represent more than 80% of the transcribed human genome. Emerging evidence suggests that microRNAs and lncRNAs regulate target genes and play an important role in biological processes and signaling pathways in malignancies, including lung cancer. In lung cancer, several tumor suppressor/oncogenic microRNAs and lncRNAs function as biomarkers for metastasis and prognosis, and thus may serve as therapeutic tools. In this review, recent work on microRNAs and lncRNAs is introduced and briefly summarized with a focus on potential biological and therapeutic applications.

## 1. Introduction

Lung cancer is the leading cause of cancer deaths among males in both developing and developed countries, and it has outpaced breast cancer as the leading cause of cancer deaths in females in developed countries [1]. Lung cancers are categorized into two main histological groups: small cell lung cancer (SCLC, 15% of all lung cancers) and non-SCLC (NSCLC, 85% of all lung cancers). NSCLCs are further subcategorized into three major histological subtypes: adenocarcinoma, squamous cell carcinoma (SqCC), and large cell carcinoma. Lung cancers are heterogeneous diseases characterized by distinct epigenetic and genetic abnormalities, even within the same histological type [2,3,4,5,6,7,8,9,10,11,12,13,14,15,16,17,18]. Although extensive efforts to develop molecular-targeted drugs are ongoing [19,20,21,22,23,24,25,26,27], no targeted therapies are available for a number of lung cancer patients. Although techniques and strategies for the early detection of lung cancer have improved, most lung cancers are still diagnosed at advanced stages. Therefore, the identification of novel diagnostic biomarkers and therapeutic strategies is a prerequisite for the management of lung cancer.

In recent years, the dysregulation of microRNAs and long non-coding RNAs (lncRNAs) has been highlighted as potential diagnostic and therapeutic tools in malignancies, including lung cancer [28,29,30,31,32,33,34,35,36,37,38,39,40,41,42,43,44,45,46,47,48,49]. Indeed, microRNAs and lncRNAs regulate a number of target genes, play an important role in lung carcinogenesis, and serve as potential diagnostic and therapeutic tools in lung cancer [37,38,39,40,41,42,43,44,45,46,47,48,49].

In this review, recent work on lncRNAs and microRNAs in lung cancer is summarized with a focus on their biological and therapeutic applications.

## 2. Non-Coding RNA

**Figure 1:**
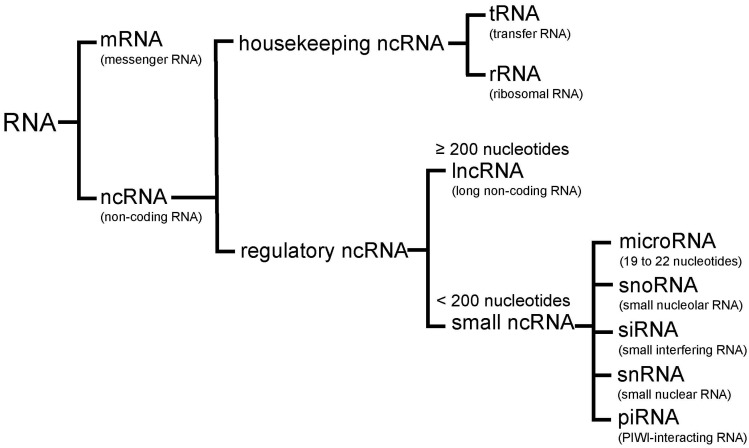
RNA categories. RNAs are divided into two major classes: messenger RNA (mRNA) and non-coding RNA (ncRNA). NcRNAs include housekeeping ncRNA, which consists of transfer RNA (tRNA) and ribosomal RNA (rRNA), and regulatory ncRNA. Regulatory ncRNAs are classified into long ncRNA (lncRNA) and small ncRNA. Small ncRNAs are subclassified into microRNA, small nucleolar RNA (snoRNA), small interfering RNA (siRNA), small nuclear RNA (snRNA), and PIWI-interacting RNA (piRNA).

Emerging evidence from the human genome sequencing projects suggests that more than 80% of the human genome is actively transcribed into RNA, even though less than 3% of the human genome encodes translated proteins [50,51]. RNAs that do not yield coding proteins are collectively referred to as non-coding RNAs (ncRNA). These ncRNAs are divided into housekeeping ncRNAs and regulatory ncRNAs. Housekeeping ncRNAs include transfer RNAs (tRNAs) and ribosomal RNAs (rRNAs). Regulatory ncRNAs are generally transcribed in a location- and time-dependent fashion. Regulatory ncRNAs can be further divided into two groups based on their size: small ncRNAs (shorter than 200 nucleotides) and lncRNAs (200 nucleotides or longer). Small ncRNAs contain microRNAs, small nucleolar RNAs (snoRNAs), small interfering RNAs (siRNAs), small nuclear RNAs (snRNAs), and PIWI-interacting RNAs (piRNAs) (Figure 1) [49,50,51].

### 2.1. MicroRNAs

MicroRNAs are small single stranded ncRNAs (19 to 22 nucleotides in length) that are highly conserved among different organisms. MicroRNAs play important regulatory roles in animals and plants by targeting messenger RNAs (mRNAs) for translational repression or degradation. MicroRNAs comprise one of the most abundant classes of gene regulatory molecules and therefore influence the output of many protein-coding genes [52]. MicroRNAs have the potential to serve as biomarkers and therapeutic tools for different cancer subtypes, as classified by origin, histology, aggressiveness, or chemosensitivity [37,38,39,40,45,46,47,48,49]. Importantly, in comparison to mRNAs, microRNAs are markedly less degraded in formalin-fixed paraffin-embedded (FFPE) samples, which are typically collected and stored in hospitals. Therefore, the easy availability of archived FFPE samples and the ability to accurately measure microRNA expression enables translation studies on microRNAs. Unlike mRNA, microRNAs exist in tissues and body fluids, such as blood and sputum. Thus, the characteristics of microRNAs support the development of liquid biopsies.

### 2.2. LncRNAs

LncRNAs are largely polyadenylated and comprise more than 200-nucleotide or longer transcripts. LncRNAs are also engaged in gene expression via transcriptional and epigenetic regulation, imprinting, splicing, and subcellular transport. Although the major mechanism of lncRNAs is the regulation of expression of neighboring genes, lncRNAs also serve as scaffold for protein–protein interactions or decoys to proteins [53]. Additionally, lncRNAs can regulate kinase functions. LncRNA *NBR2* engages a metabolic checkpoint by regulating AMP-activated protein kinase (AMPK) under energy stress [54]. Accumulating evidence suggests that lncRNAs play a role in fundamental biological functions, and dysregulation of lncRNAs contributes to cancer development, progression, and metastasis in many malignancies, including lung cancer [41,42,43,44]. Therefore, lncRNAs can be used as therapeutic targets.

Notably, lncRNAs can serve as minimally invasive and sensitive molecular markers for the screening and early diagnosis of lung cancer [55]. Peng et al. constructed a serum ncRNA panel (*miR-1254*, *miR-485-5p, miR-574-5p*, and lncRNA *MALAT1*), and tested whether the ncRNA panel could distinguish NSCLC patient samples from controls. Their results indicated that the four ncRNA panel can serve as a convenient tool for early NSCLC diagnosis [55]. LncRNAs are stable even in body fluids and show tissue-specific expression. These characteristic features make lncRNAs attractive as potential biomarkers in liquid biopsies.

## 3. Experimental Techniques to Detect MicroRNAs and LncRNAs

The golden standard for experimental techniques to detect microRNAs and lncRNAs is considered to be reverse transcriptase quantitative polymerase chain reaction (RT-qPCR). However, regarding the extraction methods, kits, controls, and quantification methods, no standards exist, which can affect the results [56]. Nonetheless, the traditional amplification method, RT-qPCR, has been well established and widely used because of its higher sensitivity, wider dynamic range, and higher precision [57]. Compared to the methods of quantifying lncRNAs, those of quantifying microRNAs have advanced. Because the traditional amplification-based method is not enough to fulfill the various requests of laboratory and clinical applications, RT-free qPCR, isothermal amplification methods, and some cross-platforms combined with amplification-based methods have been developed. Among them, the traditional RT-qPCR and cross-platforms including PCR-based arrays and next generation sequencing have been well designed and are useful [57].

## 4. MicroRNAs and Lung Cancer

**Figure 2:**
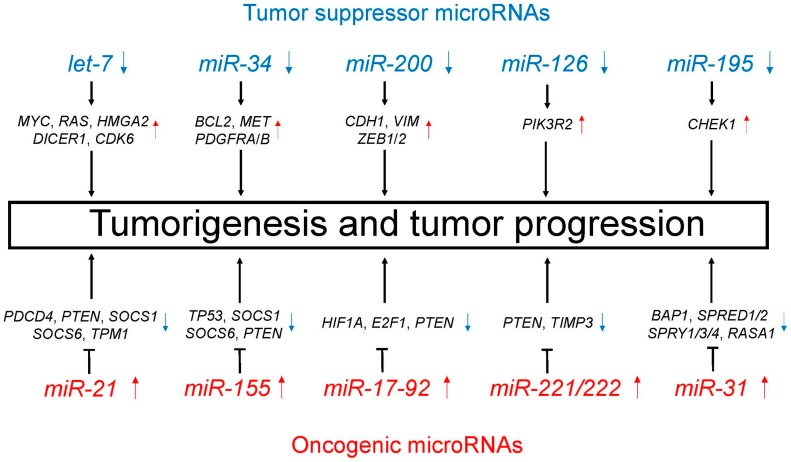
Tumor suppressor (blue)/oncogenic (red) microRNAs regulate unique target genes. This regulation by tumor suppressor/oncogenic microRNAs leads to tumorigenesis and tumor progression.

A number of studies have identified aberrant microRNA expression in lung cancer, thus supporting microRNAs involvement in lung carcinogenesis [37,38,39,40]. MicroRNAs play important regulatory roles in lung cancer, including both tumor suppression and oncogenesis. Table 1 and Figure 2 show the major tumor suppressor and oncogenic microRNAs in lung cancer. In this section, tumor suppressor and oncogenic microRNAs are introduced with suggestions on their potential utility in the diagnosis and treatment of lung cancer.

### 4.1. Tumor Suppressor MicroRNAs

#### 4.1.1. *Let-7*

*Let-7* is one of the earliest identified tumor suppressor microRNAs in lung cancer, and its decreased expression is associated with poor prognosis [58]. *Let-7* expression is reduced in adenocarcinoma in situ (AIS) when compared with adjacent normal lung tissue, indicating that *let-7* expression is decreased during the early phase of lung carcinogenesis [59]. *Let-7* inhibits the expression of oncogenes involved in cell proliferation, such as *MYC*, *RAS*, and *HMGA2* [60,61]. Interestingly, *let-7* directly down-regulates *DICER1* expression, indicating that the global production of microRNAs may be regulated by *let-7* [62]. Furthermore, *let-7* regulates the cell cycle by inhibiting *CDK6* expression [61]. Recently, Zhou et al. showed that *miR-203* played an important role in promoting the apoptosis and inhibiting the cell proliferation of lung cancer by downregulating LIN28B and upregulating *let-7* biogenesis. Their results demonstrate a novel regulatory network among *miR-203*, LIN28B, and *let-7* in lung cancer [63].

#### 4.1.2. *MiR-34*

*MiR-34* is directly transcribed by TP53, responding to DNA damage and oncogenic stress. *MiR-34* is an important component of TP53 tumor suppressor function [30]. Decreased expression of *miR-34* in lung cancer induces increased-expression of *miR-34* target genes, such as *BCL2*, *MET*, *PDGFRA*, and *PDGFRB*, which leads to TNF-related apoptosis-inducing ligand (TRAIL)-induced cell death. The down-regulation of *miR-34* upregulates *MET* and *BCL2*, which leads to cell proliferation [64,65,66]. A recent study found that tumor PD-L1 expression is regulated by TP53 via *miR-34* [67]. *MiR-34*, which is transcribed by TP53, directly binds to the PD-L1 3′ untranslated region and downregulates it. The identified TP53/*miR-34*/PD-L1 axis deserves consideration for the improvement of emerging immunotherapy.

#### 4.1.3. *MiR-200*

*MiR-200* plays a critical role in the inducement of epithelial-mesenchymal transition (EMT). Decreased expression of *miR-200* up-regulates its target genes, namely *CDH1* (also known as *E-cadherin*), *VIM* (also known as *vimentin*), *ZEB1*, and *ZEB2*, which leads to EMT as lung cancer progresses [68,69]. Zhang et al. evaluated the effect of decitabine, a DNA methyltransferase inhibitor, on TGF-β1-induced EMT in NSCLC cells, considering the involvement of the *miR-200*/ZEB axis. Decitabine reversed TGF-β1-induced EMT in NSCLC cells by downregulating ZEB1 and ZEB2 epigenetically by *miR-200*. They found that epigenetic regulation of the *miR-200*/ZEB axis is responsible for EMT induction by TGF-β1 in NSCLC cells, and that decitabine inhibits EMT in NSCLC cells via *miR-200* [70].

#### 4.1.4. *MiR-126*

The decreased expression of *miR-126* in lung cancer reduces its target *PIK3R2* and the PTEN/PI3K/AKT signaling pathway is therefore influenced, leading to the suppression of cell growth, migration, and invasion [71]. Recently, An et al. found that Matrine, which is an active component of traditional Chinese medicine, induces cell cycle arrest and apoptosis by upregulating *miR-126* in NSCLC cells [72].

#### 4.1.5. *MiR-195*

*MiR-195* expression is lower in NSCLC than in non-cancerous normal tissues and low *miR-195* expression has been associated with unfavorable overall survival of patients with NSCLC. *MiR-195* suppresses cancer growth and is associated with lower mortality in several cancers, including NSCLC. *CHEK1* is a direct target of *miR-195*, which results in decreased *CHEK1* expression in lung cancer. Up-regulation of *CHEK1* by reduced expression of *miR-195* promotes cell proliferation, migration, and invasion, and is associated with a higher overall mortality in lung cancer [73].

### 4.2. Oncogenic MicroRNAs

#### 4.2.1. *MiR-21*

*MiR-21* represents one of the most famous oncogenic microRNAs, and it is over-expressed in a number of malignancies, including lung cancer. High expression of *miR-21* predicts recurrence and higher mortality in NSCLC [74]. Furthermore, abundant *miR-21* exists in body fluids, being one of the most promising microRNAs in patients with lung cancer [49]. *MiR-21* promotes carcinogenesis via inhibition of negative regulators of RAS/MEK/ERK signaling pathway and apoptosis suppression. Over-expressed *miR-21* down-regulates the expression of *PDCD4*, *PTEN*, *SOCS1*, *SOCS6*, and *TPM1*, thus promoting cell proliferation and migration and inhibiting apoptosis [75,76,77,78]. *MiR-21* has been used as a biomarker to predict therapeutic responses to cisplatin [79]. Xu et al. conducted in vitro and in vivo experiments and demonstrated that downregulation of *miR-21* suppression increased the cisplatin sensitivity of NSCLC. Regarding radiosensitivity, downregulation of *miR-21* sensitized radio-resistant NSCLC A549 cells to ionizing radiation through the inhibition of the PI3K/AKT signaling pathway [80].

In addition, certain drugs were reported to downregulate *miR-21*. Solasodine is an aglycone of solamargine and solasonine, which are the major solasodine glycosides in eggplant. Shen et al. demonstrated that solasodine inhibited the invasion of NSCLC A549 cells via the downregulation of *miR-21* and matrix metalloprotease (MMP) expression [81]. Triptolide is isolated from Tripterygium wilfordii plants and used in traditional Chinese medicine. Lie et al. demonstrated that triptolide reduced proliferation and enhanced the apoptosis of NSCLC PC-9 cells in a *PTEN*-dependent manner by downregulating *miR-21* [82].

#### 4.2.2. *MiR-155*

As with *miR-21*, *miR-155* is an important oncogenic microRNA that has been suggested as a therapeutic target in NSCLC [74,78,83,84,85]. As with *miR-21*, *miR-155* is also a promising circulating microRNA in patients with lung cancer [49]. *MiR-155* directly targets *TP53*, whereas TP53 directly regulates the expression of *miR-155*. This *miR-155*/TP53 feedback loop is involved in chemotherapy resistance [85]. Xue et al. reported that *miR-21* and *miR-155* promote the progression of NSCLCs, in part by downregulating *SOCS1*, *SOCS6*, and *PTEN*, all of which are tumor suppressor genes. Thus, the combined inhibition of *miR-21* and *miR-155* may improve the treatment of NSCLCs [78].

#### 4.2.3. *MiR-17-92*

The *miR-17-92* intronic cluster comprises seven different microRNAs (*miR-17-5p*, *miR-17-3p*, *miR-18a*, *miR-19a*, *miR-19b-1*, *miR-20a*, and *miR-92*) and resides in intron 3 of the *C13orf25* gene at 13q31.3 [86]. Overexpression of the *miR-17-92* cluster with occasional gene amplification plays a role in the lung carcinogenesis, especially SCLC [86]. The *MiR-17-92* cluster down-regulates *HIF1A*, *E2F1*, and *PTEN*, which leads to cell proliferation and cancer progression [87,88]. Matsubara et al. reported that antisense oligonucleotides against *miR-17-5p* and *miR-20a* within the *miR-17-92* cluster induced apoptosis in lung cancers overexpressing *miR-17-92* [87]. Recently, Li et al. found that FLI1, which is an Ets transcription factor family member and known as a major driver of hematological malignancies, plays an important role in tumor progression in SCLC. Furthermore, they uncovered FLI1 as a critical driving factor that promotes cancer growth in SCLC through the *miR-17-92* pathway [89]. FLI1 and *miR-17-92* may serve as promising therapeutic targets to improve the treatment of SCLCs.

#### 4.2.4. *MiR-221/222*

Both *miR-221* and *miR-222* have an identical seed sequence and are predicted to have overlapping targets. *MiR-221/222* is associated with tyrosine kinase inhibitor (TKI)-resistant NSCLCs. Garofalo et al. reported that TKI-resistance was overcome by anti-*miR-221/222* and anti-*miR-30c*, which recovered BCL2L11 expression and increased the gefitinib sensitivity of NSCLCs, thus providing a microRNA-mediated therapeutic approach for TKI-resistant NSCLCs [90]. *MiR-221/222* influences lung carcinogenesis by down-regulating *TIMP3* and *PTEN* tumor suppressor genes [91,92]. Up-regulated *miR-221/222* promotes cell migration and suppresses apoptosis by targeting *TIMP3* and *PTEN*.

#### 4.2.5. *MiR-31*

*MiR-31* targets BAP1, which is a necessary nuclear-located deubiquitinating enzyme that acts as a tumor suppressor in lung cancer. Increased expression of *miR-31* reduced the BAP1 expression, leading to cell proliferation and suppressed apoptosis [93]. Edmonds et al. reported that *miR-31* directly downregulated six negative regulators of the RAS/MAPK signaling pathway (*SPRED1*, *SPRED2*, *SPRY1*, *SPRY3*, *SPRY4*, and *RASA1*) and promoted mutant *KRAS*-mediated oncogenesis [94].

Although many studies suggest that *miR-31* is an oncogenic microRNA, there are reports suggesting that *miR-31* functions as a tumor suppressor in lung cancer [95,96]. Xu et al. reported that the downregulation of *miR-31* enhanced lung cancer proliferation and migration by upregulating HuR, an RNA binding protein [95]. Okudela et al. reported that restoration and knockdown of *miR-31* in lung cancer cell lines attenuated their growth activities and enhanced oncogenic phenotypes, respectively, suggesting that *miR-31* acts as a tumor suppressor [95,96]. Further research is required to determine whether *miR-31* plays a pleiotropic role in individual tumors.

## 5. LncRNAs and Lung Cancer

**Figure 3:**
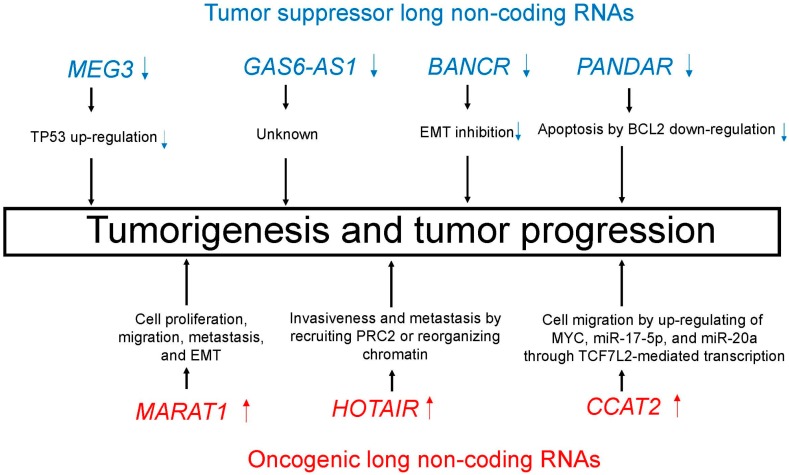
Tumor suppressor (blue)/oncogenic (red) long non-coding RNAs (lncRNAs) induce important processes that lead to tumorigenesis and tumor progression.

Emerging evidence suggests the aberrant expression of lncRNAs in lung cancer, thus indicating a role for lncRNAs in lung carcinogenesis [41,42,43,44]. Many lncRNAs play critical regulatory roles in lung cancer as tumor suppressor or oncogenic lncRNAs. Table 2 and Figure 3 summarize major tumor suppressor and oncogenic lncRNAs in lung cancer.

### 5.1. Tumor Suppressor LncRNAs

#### 5.1.1. *MEG3*

*MEG3*, which is located on 14q32.2, is a maternally expressed imprinted lncRNA found in a variety of normal tissues. *MEG3* expression was reduced in several cancers, and up-regulation of *MEG3* inhibits tumor growth [97]. Lu et al. reported that the expression of *MEG3* was down-regulated in NSCLCs when compared to adjacent normal lung tissues; furthermore, decreased expression of *MEG3* was associated with a relatively poor prognosis [98]. *MEG3* inhibits NSCLC cell proliferation and induces apoptosis by up-regulating TP53 expression [97,98]. As with NSCLC, downregulated *MEG3* in breast cancer also regulates proliferation, migration, and invasion by depending on the transcriptional activity of TP53 [99].

#### 5.1.2. *GAS6-AS1*

*GAS6-AS1*, which is located on chromosome 13q34, is transcribed antisense to the *GAS6* gene. Down-regulation of *GAS6-AS1* promotes cancer progression in several malignancies, including lung cancer [100]. Han et al. demonstrated that *GAS6-AS1* expression is lower in NSCLC than adjacent non-cancerous normal tissue and suggested downregulation of *GAS6-AS1* as an independent biomarker for higher overall mortality in NSCLC patients [100]. Because the molecular mechanisms of *GAS6-AS1*-mediated NSCLC progression still remain elusive, further mechanical studies should be required.

#### 5.1.3. *BANCR*

*BANCR* mediates cell growth by regulating cell-growth arrest, leading to the reduction of cancer incidence. The expression of *BANCR* is significantly down-regulated in NSCLC tissues when compared to adjacent non-cancerous normal lung tissues, and the lower *BANCR* expression has been associated with higher mortality in NSCLC patients [101]. Although the mechanical explanation why *BANCR* suppresses invasiveness and metastasis of NSCLCs remains unclear, it is likely associated with EMT inhibition. Loss of *BANCR* expression reduces CDH1 (also known as E-cadherin) expression and induces CDH2 (also known as N-cadherin), VIM (also known as vimentin), and MMPs [101]. Thus, *BANCR* is also a potential target of lncRNA-mediated therapeutics.

#### 5.1.4. *PANDAR*

*PANDAR*, which is located on chromosome 6q21.2, is a tumor suppressor lncRNA. Han et al. reported that decreased expression of *PANDAR* was associated with higher overall mortality in NSCLC patients. A direct transcription target of *PANDAR* includes TP53 in NSCLC cells, and *PANDAR* affects cell apoptosis by regulating BCL2 [102]. Because *PANDR* is a direct transcriptional target of TP53 in NSCLC, overexpression of *PANDR* could inhibit the proliferation of NSCLC cells.

### 5.2. Oncogenic LncRNAs

#### 5.2.1. *MALAT1*

*MALAT1*, also known as *NEAT2*, is a promising lncRNA, and is a candidate of biomarker in liquid biopsy for the diagnosis of NSCLCs. Furthermore, high *MALAT1* expression in FFPE specimens indicates a higher mortality in NSCLCs and experimentally promotes cell proliferation, migration, metastasis, and EMT [103,104,105]. One possible mechanistic explanation for the oncogenic activity of *MALAT1* is that *MALAT1* participates in aberrant alternative splicing, which results in the dysregulated expression of genes, including B-MYB transcription factor [106]. Recently, Wang et al. conducted a follow-up study for 538 patients of NSCLC, and genetic variant rs3200401 in *MALAT1* was then genotyped among this population. They demonstrated that the rs3200401 T allele located on the lncRNA *MALAT1* was associated with lower mortality for patients with advanced lung adenocarcinoma [107].

#### 5.2.2. *HOTAIR*

*HOTAIR* is a lncRNA, which is located downstream, in the antisense direction, of *HOXC12* gene [108]. *HOTAIR* promotes the invasiveness and metastasis of several cancer types by recruiting PRC2 or chromatin reorganization [109]. Additionally, *HOTAIR* has been reported to participate in chemoresistance to cisplatin in lung adenocarcinoma [110]. Liu et al. also reported that elevated *HOTAIR* expression was associated with cisplatin resistance in NSCLC, and showed that *HOTAIR* expression was directly related to KLF4 expression, suggesting a new therapeutic target for drug-resistance patients with NSCLC [111].

#### 5.2.3. *CCAT2*

*CCAT2* is a novel lncRNA and its overexpression is associated with a number of cancers, including NSCLC. *CCAT2* is highly expressed in NSCLCs, especially adenocarcinoma. According to a study using NSCLC cell lines by Qiu et al. [112], reducing *CCAT2* expression by siRNA inhibits tumor proliferation and invasiveness in NSCLC cells. *CCAT2* promotes cell migration by up-regulating *MYC*, *miR-20a*, and *miR-17-5p* through TCF7L2-mediated transcription in cell lines [113]. Recently, Zhao et al. showed that *CCAT2* promotes tumorigenesis by overexpressing of Pokemon (also known as ZBTB7A) and suggested that the potential mechanism might relate to the Pokemon-related gene CDKN1A (also known as p21) [114].

## 6. Conclusions and Future Directions

In this review, recent studies of microRNAs and lncRNAs in lung cancer are introduced with a focus on potential biological and therapeutic applications. Accumulating evidence suggests that microRNAs and lncRNAs represent very promising biomarkers in patients with NSCLC and will be markedly useful in non-invasive screening methods. MicroRNA- or lncRNA-mediated therapy for patients with lung cancer is also very promising. Further studies and clinical trials are needed to assess microRNA or lncRNA profiles as diagnostic markers and conduct microRNA- and lncRNA-based therapies in clinical practice.

## Figures and Tables

**Table 1:** Tumor suppressor and oncogenic microRNAs in lung cancer.

MicroRNA	Expression	Affected Biological Processes and Target Genes
Tumor suppressor microRNAs		
*let-7*	Decreased	i) Cell proliferation (*MYC*, *RAS*, *HMGA2*) [60,61] ii) MicroRNA biogenesis (*DICER1*) [62] iii) Cell cycle regulation (*CDK6*) [61]
*miR-34*	Decreased	TRAIL-induced cell death and cell proliferation (*BCL2*, *MET*, *PDGFRA*, *PDGFRB*) [64,65,66]
*miR-200*	Decreased	Promotion of EMT and metastasis (*CDH1*, *VIM*, *ZEB1*, *ZEB2*) [68,69]
*miR-126*	Decreased	Cell proliferation, migration, and invasion through PTEN/PI3K/AKT pathway (*PIK3R2*) [71]
*miR-195*	Decreased	Cell proliferation, migration, and invasion (*CHEK1*) [73]
Oncogenic microRNAs		
*miR-21*	Increased	Cell proliferation, migration, and apoptosis (*PDCD4*, *PTEN*, *SOCS1*, *SOCS6*, *TPM1*) [75,76,77,78]
*miR-155*	Increased	Resistance to chemotherapy (*TP53*) [85] Cell proliferation and apoptosis (*SOCS1*, *SOCS6*, *PTEN*) [78]
*miR-17-92*	Increased	Carcinogenesis and cell proliferation (*HIF1A*, *E2F1*, *PTEN*) [87,88]
*miR-221/222*	Increased	Cell migration and apoptosis (*PTEN*, *TIMP3*) [91,92]
*miR-31*	Increased	Cell proliferation and apoptosis (*BAP1*) [93] Promotion of KRAS/MAPK signaling (*SPRED1*, *SPRED2*, *SPRY1*, *SPRY3*, *SPRY4*, *RASA1*) [94]

EMT, epithelial-mesenchymal transition. TRAIL, TNF-related apoptosis-inducing ligand.

**Table 2:** Tumor suppressor and oncogenic long non-coding RNAs (lncRNAs) in lung cancer.

MicroRNA	Chromosome	Biological Processes
Tumor suppressor lncRNAs		
*MEG3*	14q32.2	TP53 up-regulation [97,98]
*GAS6-AS1*	13q34	Unknown
*BANCR*	9q21.12	EMT inhibition [101]
*PANDAR*	6q21.2	Apoptosis by BCL2 down-regulation [102]
Oncogenic lncRNAs		
*MALAT1*	11q13.1	Cell proliferation, migration, metastasis, and EMT [105]
*HOTAIR*	12q13.13	Invasiveness and metastasis by recruiting PRC2 or reorganizing chromatin [109]
*CCAT2*	8q24.21	Cell migration by up-regulating of *MYC*, *miR-17-5p*, and *miR-20a* through TCF7L2-mediated transcription [113]

EMT, epithelial-mesenchymal transition; lncRNA, long non-coding RNA.
